# Surgery for Radiologically Normal-Appearing Temporal Lobe Epilepsy in a Centre with Limited Resources

**DOI:** 10.1038/s41598-020-64968-4

**Published:** 2020-05-18

**Authors:** Muhamad Thohar Arifin, Yuriz Bakhtiar, Erie B. P. S. Andar, Happy Kurnia B., Dody Priambada, Ajid Risdianto, Gunadi Kusnarto, Krisna Tsaniadi, Jacob Bunyamin, Ryosuke Hanaya, Kazunori Arita, Aris Catur Bintoro, Koji Iida, Kaoru Kurisu, Rofat Askoro, Surya P. Briliantika, Zainal Muttaqin

**Affiliations:** 1 Department of Neurosurgery, Faculty of Medicine, Diponegoro University, Jl Prof. Soedarto, Tembalang, Semarang, Jawa Tengah, Indonesia; 2 Department of Neurosurgery, Graduate School of Medical and Dental Sciences, Kagoshima University, Kagoshima, Japan; 3 Department of Neurology, Faculty of Medicine, Diponegoro University, Jl Prof. Soedarto Tembalang, Semarang, Jawa Tengah, Indonesia; 4 Department of Neurosurgery, Graduate School of Biomedical and Health Sciences, Hiroshima University, Hiroshima, Japan

**Keywords:** Medical research, Neurology

## Abstract

Approximately 26–30% of temporal lobe epilepsy (TLE) cases display a normal-appearing magnetic resonance image (MRI) leading to difficulty in determining the epileptogenic focus. This causes challenges in surgical management, especially in countries with limited resources. The medical records of 154 patients with normal-appearing MRI TLE who underwent epilepsy surgery between July 1999 and July 2019 in our epilepsy centre in Indonesia were examined. The primary outcome was the Engel classification of seizures. Anterior temporal lobectomy was performed in 85.1% of the 154 patients, followed by selective amygdalo-hippocampectomy and resection surgery. Of 82 patients (53.2%), Engel Class I result was reported in 69.5% and Class II in 25.6%. The median seizure-free period was 13 (95% CI,12.550–13.450) years, while the seizure-free rate at 5 and 12 years follow-up was 96.3% and 69.0%, respectively. Patients with a sensory aura had better seizure-free outcome 15 (11.575–18.425) years. Anterior temporal lobectomy and selective amygdala-hippocampectomy gave the same favourable outcome. Despite the challenges of surgical procedures for normal MRI TLE, our outcome has been favourable. This study suggests that epilepsy surgery in normal MRI TLE can be performed in centres with limited resources.

## Introduction

Temporal lobe epilepsy (TLE) is still the most widely known focal epilepsy type and it can often become drug-resistant. The most frequent related pathology is hippocampal sclerosis and this has a favourable postoperative result^1^. However, 26–30% of all cases show a normal-appearing result in magnetic resonance imaging (MRI). This makes it difficult to determine its epileptogenic focus and is associated with a lower rate of seizure freedom especially in comparison to the positive MRI patients^2–4^. Additionally, there can be challenges in the further evaluation and treatment of normal MRI TLE patients in resource-limited settings. Surgical management tends to be challenging due to the non-specificity of the location and the uncertainty associated with the positive MRI^2–4^. The seizure-free rate in patients treated with surgery is significantly higher than those treated conservatively^5^.

The distinction between lesional and normal-appearing TLE is based on abnormal hippocampal signalling and the severity of neuronal loss observed on histopathological examination. Normal cases are thought to be a separate entity, rather than a ‘milder form’^6^. Reaching a decision for surgical treatment can be challenging because the determination of the epileptogenic focus requires advanced examination which is often expensive and time-consuming, especially in a centre with limited resources. The side of surgery for lesional TLE is determined by seizure semiology, MRI and scalp electroencephalography (EEG) recording.

The determination of the epileptic zone requires further diagnostic measures, including the scalp and intracranial EEG, functional MRI (fMRI), magnetic resonance spectroscopy, fluoro‐2‐deoxy‐d‐glucose positron emission tomography (FDG‐PET) and single-photon emission computed tomography.

This research reviews cases of normal-appearing temporal MRI subjected to epilepsy surgery at the Semarang epilepsy centre, Indonesia. Semarang is the only epilepsy centre in Indonesia and thus serves a population of ¼ billion. At the time of the study, there was only one FDG-PET facility in Jakarta. At present, there are only 3 EEG video centres in Indonesia and before 2010 we used a separate video camera and synchronised time manually with EEG. We obtained an EEG video in 2011. EEG electrode monitoring and subdural EEG electrodes were donated by Japanese colleagues and teachers.

## Methods

### Study design

This was a retrospective observational study conducted at the epilepsy centre in Semarang, using data collected from the medical records of 723 surgically treated patients, between July 1999 and July 2019. This research was approved by the institution review board of Kariadi General Hospital, in accordance with the Helsinki declaration. Prior written informed consent was obtained from all patients. For patients under the age of 18 years, informed consent obtained from a parent and/or legal guardian.

### Patient selection

Patients were included if they had drug-resistant epilepsy and presented with temporal semiology, including auras (olfactory, abdominal and psychic), oral or manual automatism and postictal amnesia on observation. Furthermore, patients meeting these criteria were all screened and had routine preoperative examinations, including an MRI (1.5 Tesla, Siemens). Patients in whom hyperintense signals and/or atrophy were not shown in T2-weighted images in the temporal lobe were included in this study. Patients were excluded if they had other pathological findings within the temporal lobe. Patients also had scalp EEG (since 1999) or video-monitoring EEG (since 2011), which was placed according to the International 10–20 system. The data collected included dates of surgery, seizure recurrence and last follow-up, age at seizure onset and at surgery, gender, side of surgery, seizure frequency, type of surgery and preoperative fluorodeoxyglucose positron emission tomography (FDG-PET) metabolism. At least 1 year of postsurgical follow-up was required for inclusion in this study.

### Imaging protocols

MRI studies were performed using a 1.5-T MRI machine (Siemens, Erlangen, Germany), observing the protocols for coronal section T1-weighted, T2-weighted and fluid-attenuated inversion recovery sequences. In addition, the hippocampi were assessed from the hyperintense signals in T2-weighted images or atrophy, and the images were further evaluated by neuroradiologists, epileptologists and epilepsy surgeons. A team consensus was required for a brain MRI to be classified as normal. Additional functional neuroimaging studies with FDG-PET were obtained for patients when a consensus regarding the lateralisation of the semiology was not achieved. Patients who could afford this were referred for examination at hospitals with FDG-PET facilities. Such hospitals were in Jakarta and Singapore, which are far from our centre.

### Surgical considerations

All cases were discussed with the epilepsy team and the decision to operate was based on the judgement of an epilepsy neurosurgeon, epileptologist and neuropsychologist. A decision would be made at a meeting whether to perform additional evaluation or proceed with surgery. The type of surgery, e.g., anterior temporal lobe (ATL), selective amygdala-hippocampectomy (SAH), or resection was chosen based on clinical judgement and the results of preoperative evaluations. For additional evaluation, subdural electrodes (SDEs) were implanted in several subjects, based on the following indications: (1) to confirm the temporal ictal zone in non-localising scalp EEG or inconsistency with seizure semiology; (2) to exclude bi-temporal, pseudo-temporal, or temporal plus epilepsy in instances where the nuclear imaging raised suspicion (3) to assess the possibility of hippocampal sparing or perform preoperative functional mapping when the localisation was assumed to be near eloquent areas. Moreover, intraoperative electrocorticography (ECoG) was performed in several cases, in an attempt to map the extent of epileptogenic cortex resection or to spare eloquent areas of the cortex. Subdural electrodes were donated by our Japanese affiliate epilepsy centre.

Generally, ATL was reserved for patients with the epileptogenic zone in the non-dominant lobe or for dominant lobe patients with mental retardation, while SAH was excluded for the epileptic zone in the dominant lobe in patients with a good IQ. Furthermore, ATL was conducted by removing the temporal neocortex, including the hippocampus to the level of the superior colliculus. This was extended to about 4 to 4.5 cm, in dominant lobes and 5 to 5.5 cm in non-dominant lobes, from the anterior temporal fossa along the medial temporal gyrus. The SAH involved resecting the hippocampus, parahippocampal gyrus and amygdala along the line connecting the choroidal point of the lateral ventricle temporal horn to the M1 division of the middle cerebral artery, either via the transsylvian or transsulcal approach. The extent of resection was influenced by the result of invasive EEG monitoring.

### Outcomes

Postsurgical follow-up of at least 1 year was required for inclusion in this study. The primary outcome was the Engel classification of seizure. The frequency of seizure was documented based on the most recent visit, posted questionnaire or telephone interview. The categorisation was defined as Class I, free of disabling seizures; Class II, rare disabling seizures/almost seizure-free; Class III (worthwhile improvement); and Class IV (no worthwhile improvement). We divided the cohort into two broad categories, one as ‘seizure-free (1a)’ with or without AEDs during the whole period of postoperative follow-up as ‘good outcome’ and seizures of any type at any time after surgery as ‘poor outcome’.

### Statistical analysis

Pearson’s chi-square test and Fisher’s exact test were used to compare the characteristics between seizure-free patients and patients with seizures. Univariate logistic regression analysis was used to assess the prognostic importance of aura sub-type and seizure type for postoperative seizure outcome. Significant variables in the preliminary analysis were included in the Cox proportional hazards regression model. A Kaplan–Meier plot was used to analyse the outcome (seizure-free chance in a year) for each variable. P < 0.05 was considered statistically significant. A computerised statistic (IBM SPSS 24) was used to perform all analyses.

## Results

Patient characteristics, initial frequency and seizure semiology are described in Table 1. A total of 154 patients with normal-appearing temporal MRI were enrolled, and the average time since the onset of epilepsy was 11.5 ± 6.4 years, with the mean age at surgery of 23.1 ± 8.9 years. Based on semiology, most patients had focal impaired awareness seizure (FIAS; 70.7%), with a frequency of less than 12 episodes per month (88.9%), and over half of the study population (69.5%) experienced auras of various types (Table 1).

**Table 1:** Characteristics of temporal lobe epilepsy patients with ‘normal-appearing’ MRI.

Characteristics	Number (%) (n = 154)
Demography	
Male/Female	91 (59.1)/63 (40.9)
Mean age at surgery	23.1 ± 8.9
Mean onset of seizure	11.5 ± 6.4
Mean duration until surgery	11.7 ± 7.2
Seizure Frequency	
Less than 12 episode/month	137 (88.9)
12 episodes or more/month	17 (11.1)
Seizure Semiology	
FAS	1 (0.6)
FIAS	109 (70.8)
FIAS to GTCS	44 (28.6)
Aura	
Absent/Present	47 (30.5)/107 (69.5)
Aura Type	
Autonomic	45 (29.2)
Sensory	14 (9.1)
Mental affective	33 (21.4)
Multiple	15 (9.7)
Preoperative AEDs	
Mean daily consumption	2.2 ± 0.9

FAS = focal aware seizure; FIAS = focal with impaired awareness seizure; GTCS = generalised tonic–clonic seizure; AED = anti-epileptic drugs.

Mean age at onset, age at surgery and duration of epilepsy were compared in the seizure and seizure-free group. Mean ages at onset in the seizure and seizure-free group were 12.114 + 5.406 years and 10.591 + 5.786 years, respectively (*p* = 0.275). Mean ages at surgery in the seizure and seizure-free group were 22.416 + 8.032 years and 22.122 + 9.363 years, respectively (*p* = 0.894). Mean duration of epilepsy in the seizure and seizure-free group was 10.301 + 4.749 and 11.717 + 7.690 (*p* = 0.406), respectively (Table 2). There were no differences in the mean age at onset, age at surgery and duration of epilepsy between the seizure and seizure-free groups.

**Table 2:** Comparison of means of age at onset, age at surgery and length of diagnosis to postoperative seizure-free outcome.

Status of Seizure in Final Follow-up		Length of Diagnosis in Years (SD)	*p*
Age at onset	Seizure	12.114 (5.406)	0.275
	Seizure-free	10.591 (5.786)	
Age at surgery	Seizure	22.416 (8.032)	0.894
	Seizure-free	22.122 (9.363)	
Duration of epilepsy	Seizure	10.301 (4.749)	0.406
	Seizure-free	11.717 (7.690)	

The results of presurgical evaluations to determine the epileptogenic zone are described in Table 3. In addition, an ictal scalp EEG was performed on 67.5% of all patients, where over half demonstrated an epileptic focus on the left side (54.5%). The temporal lobe was the most frequent zone recorded (70.8%), followed by frontotemporal and frontal. A subdural EEG was implanted in 21 patients (Table 4 reports 7 representative cases), whereas 43 patients underwent an FDG-PET scan, where half of the population exhibited hypometabolic activities in the left temporal lobe (Table 3).

**Table 3:** Presurgical evaluations in temporal lobe epilepsy patients with ‘normal-appearing’ MRI.

Presurgical Evaluations	Number (%) (n = 154)
PET scan	
Total	43 (27.9)
Left hypometabolic	24 (15.6)
Right hypometabolic	16 (10.4)
Bilateral hypometabolic	1 (0.6)
No lateralisation	2 (1.3)
Scalp EEG	
Ictal	104 (67.5)
Interictal	50 (32.5)
EEG focus (side)	
Left side	84 (54.5)
Right side	43 (27.9)
Bilateral	11 (7.1)
Diffuse	4 (2.5)
Normal	12 (8.0)

**Table 4:** Subdural EEG cases in normal MRI TLE (representative cases).

No.	Sex	Age at onset/ Duration of Epilepsy (years)	Frequency of seizure (times/month)	Type of seizure	Type of aura	AED (mg/day)	Scalp EEG findings	Subdural EEG findings	Type of operation	Final outcome
1	M	7/8	2–4	FIAS	Autonomic	CBZ 600	Bilateral IEDs	Rt. Temporal IEDs	Rt. ATL	Engel Ia
2	F	11/15	10–15	FIAS	Multiple	VPA 1000, LTG 100, CLB 20	No IEDs	Rt. Temporal IDs	Rt. ATL	Engel II
3	M	19/26	4–5	FIAS	Autonomic	PHT 300	Bilateral IEDs	Rt. Temporal IDs	RT. ATL	Engel Ia
4	M	14/5	8–12	FIAS	Mental affective	CBZ 600, PHT 200, CLB 20	No IEDs	Rt. Temporal IDs	Rt. ATL	Engel II
5	M	12/33	3–4	FIAS	Multiple	CBZ 600, CLB 20	No IEDs	Lt. Temporal IDs	Lt. ATL	Engel Ia
6	F	11/9	3–4	FIAS	Multiple	OXC 600	No IEDs	Rt. Temporal IDs	Rt. ATL	Engel Ia
7	M	12/8	3–4	FIAS to GTCS	Mental affective	VPA 750, PHT 200	No IEDs	Lt. Temporal IDs	Lt. ATL	Engel Ia

M: Male, F: Female, FIAS: Focal Impaired Awareness Seizure, FIAS to GTCS: Focal Impaired Awareness Seizure to Generalised Tonic–Clonic Seizure CBZ: Carbamazepine, VPA: Valproat, LTG: Lamotrigine, CLB: Clobazam, PHT: Phenytoine, OXC: Oxcarbazepine, IEDs: Interictal Discharges, ID: Ictal Discharges, Rt: Right, Lt: Left, ATL: Anterior Temporal Lobectomy.

Most patients were operated on from the left side (54.5%), as anterior temporal lobectomy was performed in 85.1%, followed by selective amygdalo-hippocampectomy and resection. (Tables 3 and 5) Also, Electrocorticography (ECoG) was performed on 7 individuals (Table 3).

**Table 5:** Surgical approaches in temporal lobe epilepsy patients with ‘normal-appearing’ MRI.

Surgical Approaches	Number (%) (n = 154)
Surgical side	
Left side	93 (60.3)
Right side	61 (39.7)
Surgery type	
ATL	131 (85.1)
SAH	16 (10.4)
Resection	7 (4.5)
Electrocorticography (ECoG)	7 (4.5)

ATL = anterior temporal lobectomy; SAH = selective amygdalao-hippocampectomy.

Of the 154 patients who underwent operation, 72 (46.8%) were lost to follow-up. Patients who live far away from our centre were typically managed by their local neurologist and could not be contacted by phone. Therefore, we finally included 82 patients (53.2%) who had a complete medical history of seizure follow-up. Seizure outcomes were described using the Engel classification (Table 6)—Engel I: 57 patients (69.5%), Engel II: 21 (25.6%), Engel III: 3 patients (3.7%), and Engel IV: 1 patient (1.2%).

**Table 6:** Postoperative seizure outcomes.

Seizure Outcomes	Number (%) (n = 82)
Engel Class	
I	57 (69.5)
II	21 (25.6)
III	3 (3.7)
IV	1(1.2)

A Kaplan–Meier plot was used to illustrate the chances of postoperative seizure freedom after surgery. Engel Class was used to determine the seizure-free criteria.

### Differences in anterior temporal resection versus selective outcomes

Median seizure-free time for ATL was 14 (13.139–14.861) years and for SAH was 11 (8.171–13.829) years, with no statistical difference (p = 0.519). Both ATL and SAH resulted in the same favourable outcomes (Table 7).

**Table 7:** Median seizure-free time for (Type of operation, aura, seizure and overall subjects).

Variable		Median Survival Time Year (95% CI)	*p*
Type of Operation	ATL	14 (13.139–14.861)	0.519
	SAH	11 (8.171–13.829)	
Type of Aura	Autonomic	13 (11.539–14.461)	0.013^*^
	Sensory	15 (11.575–18.425)	
	Mental Affective	10 (5.011–14.989)	
	Multiple	13 (12.487–13.513)	
Type of Seizure	FIAS	13 (12.397–13.603)	0.826
	FIAS to GTCS	13 (11.352–14.648)	
Overall Median Survival Time for Seizure Freedom		13 (12.550–13.450)	

*p < 0.05, statistically significant different each sub-variable.

### Aura, type of seizure and time of follow-up: effect on outcome

Median seizure-free time for autonomic aura was 13 (11.539–14.461) years, for sensory aura, 15 (11.575–18.425) years, and for mental and affective auras, 10 (5.011–14.989) years and 13 (12.487–13.513) years, respectively (p = 0.013). Sensory aura had the better seizure-free outcome after operation (Table 7).

Median seizure-free time for FIAS was 13 (12.397–13.603) years and for FIAS to generalised tonic–clonic seizure (GTCS) was 13 (11.352–14.648) years. There was no significant difference (*p* = 0.826). Both FIAS and FIAS to GTCS had the same postoperative outcome (Table 7).

**Figure 1:**
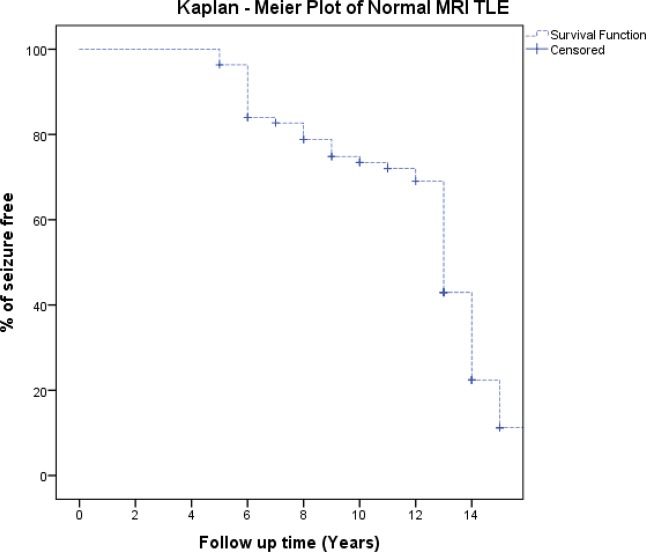
Kaplan–Meier Plot on the chances of postoperative seizure-free period in the follow-up years.

The median seizure-free time for all patients who underwent epilepsy operation was 13 (95% CI: 12.550–13.450) years. During follow-up, all patients remained seizure-free for 5 years. A total of 96.3% of patients showed a tendency towards being seizure-free within 5 years of follow-up, and 69.0% of patients remained seizure-free at the 12-year follow-up (Table 8, Fig. 1).

**Table 8:** Time between surgery and seizure-free status.

Time since surgery (years)	5	6	7	8	9	10	11	12
Seizure-free chance (%)	96.3	84.0	82.7	78.8	74.8	73.4	72.0	69.0

## Discussion

Over 20 years, 154 TLE patients were operated on because of normal-appearing MRI in our centre. Of these 82 patients completed the study, 57 patients (69.5%) remained seizure-free until the final follow-up.

Other authors have reported an outcome spanning from 41% to 76% after a minimum of a 1-year follow-up period^7–11^. With limited resources, our results were acceptable in achieving seizure-free status. This difference can be caused by the phenomenon of ‘running down’, defined as the late remission of postsurgical seizures, described in 3.2–20% of TLE cases^12,13^, where patients may experience several seizures per month during the running down interval but typically achieve a seizure-free state within 2 years^14^.

Among the factors affecting seizure outcomes, a major negative predictor was a non-lesional MRI^15,16^. Although the management of normal-appearing MRI TLE is challenging, we achieved favourable postoperative outcomes. Advances in imaging technology have resulted in the change of several normal-appearing TLE cases into the lesional category. In addition, the sensitivity of 1.5 T MRI in detection was between 82% and 86%^17^, although several studies have documented an increased value at higher magnetic fields (3 T) when visualising structural lesions^18,19^. Another study compared the initial negative MRI with images obtained using higher magnetic fields and obtained a positive result in 21% of the cases, half of which were considered potentially epileptogenic lesions^20^.

Our results showed that there were no differences in the mean age at onset, age at surgery and duration of epilepsy between the seizure and seizure-free groups. A meta-analysis of normal-appearing TLE cases found that a longer duration of epilepsy (>20 years) and ictal or interictal electroencephalographic anomalies precisely localised in the ipsilateral temporal lobe were significantly related to a higher rate of seizure freedom^1^. However, that study reported a mean duration of 11.7 ± 7.2 years, where 70.8% of the population exhibited an ipsilateral temporal lobe epileptogenic focus. Conversely, age at surgery, sex, PET localisation and the assessment side interestingly had no significant association with the seizure outcomes^1^.

Seizure auras occur in several patients with TLE and often exhibit features that are relatively specific for TLE but few are of lateralising significance. However, automatisms often have lateralising significance. Careful study of seizure semiology remains invaluable in addressing the search for the seizure onset zone^21,22^. Our results showed that the presence of a sensory aura predicted a better seizure-free outcome following operation compared with other types of aura.

It remains debatable whether SAH or standard ATL is the most effective approach concerning seizure outcome, quality of life and memory. No significant difference was observed in terms of therapeutic effects between SAH and ATL in TLE treatment^23,24^. As reported in this study, ATL and SAH had the same outcome.

Further, 43 of our cases underwent an FDG-PET scan, and half of the population exhibited hypometabolic activities in the left temporal lobe. We selected this protocol when a consensus regarding the lateralization of the semiology was not achieved because of inconsistent result of semiology, and scalp EEG. Patients who could afford this were referred for examination at hospitals with FDG-PET facilities. Such hospitals were in Jakarta and Singapore, which are far from our centre. FDG-PET is an accurate noninvasive method in lateralising the epileptogenic focus in TLE, particularly in patients with normal or equivocal MRIs or non-lateralized EEG monitoring^25^.

There were some limitations to our study. First, it was a single-centre study and there might be some data constraints. To better assess the outcome of normal MRI TLE surgery in a centre with minimal resources, a multicentre analysis with a greater number of patients is needed. Second, this sample had a high dropout rate (46.8%) owing to the number of patients lost to follow-up; this was primarily because they lived too far from our centre.

## Conclusion

Despite the challenges of the surgical procedures for normal MRI TLE, our outcomes are favourable. Compared with others, sensory aura had a better seizure-free result. ATL and SAH offer the same result. Our studies suggest that epilepsy surgery in normal MRI TLE cases can be performed in a centre with limited resources.
